# Analysis of Early Neurovascular Complications of Pediatric Supracondylar Humerus Fractures: A Long-Term Observation

**DOI:** 10.1155/2017/2803790

**Published:** 2017-03-07

**Authors:** Ryszard Tomaszewski, Artur Wozowicz, Paulina Wysocka-Wojakiewicz

**Affiliations:** Department of Pediatric Traumatology and Orthopedy and Department of Pediatric Surgery and Urology, Silesian Medical University, Katowice, Poland

## Abstract

*Purpose*. Analysis of early vascular and nerve complications of supracondylar humerus fractures in children.* Material and Methods*. 220 children hospitalized in the Pediatric Trauma-Orthopedic Department in the years 2004–2014. The group consisted of 143 males and 77 females.* Results*. Acute neurovascular complications occurred in 16.81% of patients with displaced supracondylar fracture (37 children). Nerve damage was found in 10% of patients with displaced fracture (22 children). The most injured nerve was median nerve; this complication occurred in 15 patients (68.18%). The total nerve function returned after average of 122 days (0–220 days after surgery). Symptoms of vascular injury occurred in 7.7% children with displaced fracture (17 children).* Conclusions*. (1) In children with supracondylar fracture the most often injured nerve is median nerve. (2) The incidence of vascular and nerve complications positively correlates with the progression of fracture according to Gartland classification.

## 1. Introduction

Supracondylar fracture of the humerus is one of the most common injuries in children. It represents about 16% of all pediatric fractures [1–3] and over 60% of fractures of the elbow in children [4–7]. The most common mechanism of fracture is usually extension-type fracture, when a child falls on the outstretched hand with the elbow in full extension with abduction in the scapular-shoulder join [8]. This type of supracondylar fracture accounts for 97–99% [4, 9–12]. These fractures are often accompanied by serious neurovascular complications [7]. Because of the nature of trauma which is closely associated with the children's age and the large number of patients treated conservatively or surgically each year in our hospital, we analysed cases treated between 2004 and 2014. In the group of studied patients there were children who developed neurovascular complications immediately after injury or as a result of repositioning of the fracture. Based on the research and the observation of ambulatory treatment we monitored the natural history of complications and the possible need for reoperation in cases of primary treatment failure.

## 2. Materials and Methods

The study included 220 children hospitalized in the Department of Orthopedic Traumatology due to supracondylar fracture of the humerus in the years 2004–2014. Mean age of patient's was 7.9 years (from 3 months to 16 years). There were 143 boys (65%) and 77 girls (35%). Extension-type fracture was noticed in 98% of patients and 78% of injuries affected the left side.

This is a retrospective study. Patients data were obtained from medical records of the Hospital's Emergency Department, Trauma-Orthopedic Department and Orthopedic Dispensary.

On admission to the hospital each child with suspected supracondylar humerus fracture had examination of the brachial artery's pulse, radial and ulnar artery, capillary refill time, and pulse oximetry of second finger. Neurological examination in the area of innervation of the radial nerve, ulnar nerve, and median nerve was performed.

Before and after reduction of a fracture in each patient radiograph of the elbow in anteroposterior (AP) and lateral projections was performed. To assess the degree of fracture the scale of Gartland modified by Leitch has been used [4].

Patients were treated as follows. Nondisplaced fractures were managed conservatively by immobilization in a plaster cast, displaced fractures by closed reduction, and percutaneous Kirschner wire fixation with two or three lateral divergent wires. In the case of 4 patients it was necessary to perform percutaneous pinning with two crossed K-wires (one inserted through the lateral condyle and another through the medial condyle). After hospitalization a further inspection was carried out in our Orthopedic Department and Dispensary.

## 3. Results

Acute neurovascular complications occurred in 16,81% of hospitalized patients with supracondylar fracture (37 children). All occurred in displaced fractures and responding II–IV degrees according to the modified Gartland classification.

**Figure 1:**
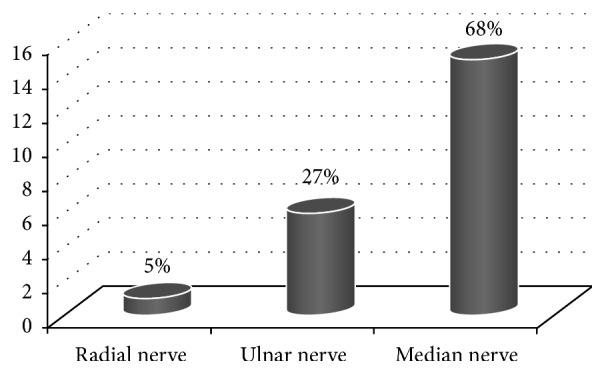
Incidence of nerve injury.

Nerve damage was found in 10% of patients with displaced fracture (22 children). The most injured nerve was median nerve; this complication occurred in 15 patients (68%). From these patients 5 cases with damage of anterior interosseous nerve were selected (the pseudoanterior interosseous nerve syndrome) [13] and 6 patients presented damage of the ulnar nerve, and radial nerve injury occurred in 1 child, which accounted for 27% and 5% of all damage to the nerves. The frequency of recorded neural structures is illustrated on Figure 1.

Symptoms with neurologic injury in 20 children resolved spontaneously. In 1 case open reduction was essential and ulnar nerve was released 2 months after trauma and in 1 case 1 month after fracture median nerve release was done. The total nerve function returned after average of 122 days.

Symptoms of vascular injury occurred in 7.7% of patients with displaced fracture (17 children). In 13 patients (76%), pulse and correct blood oxygen saturation measured on the second finger with pulse oximeter returned immediately after fracture reposition. The Doppler ultrasound was used in all cases and confirmed correct blood circulation in brachial artery.

One patient because of symptoms of poor blood supply to the limbs and no pulse return after reduction underwent reconstruction caused by entrapment of the brachial artery in one day after the reposition of the fracture. This patient also experienced reduction of sensation of the 2nd and 3rd finger, as the effects of the median nerve injury, which then disappeared after 10 days. The 2 patients, despite the return of the pulse, also required revision of the brachial artery because of the thrombosis (2 hours and 15 hours after fracture reposition). In 1 case with Gustilo III C fracture the reconstruction of the brachial artery was made using saphenous vein graft.

Each of these patients with the vascular symptoms (except for Gustilo IIIC fracture) after fracture reposition (less than 1 hour after surgery) have been examined using Doppler ultrasound. In 14 patients the Doppler exam was normal; in 3 cases the flow was reduced to 50%. Angiography was made in 2 cases. In follow-up until today, there is no unequal and asymmetrical length of the forearm and hand. One patient presents a moderate cold intolerance symptoms occurring within forearm and hand area.

Five children (2%) presented a combined neurovascular injury, in 1 child ulnar nerve injury and brachial thrombosis occurred and 4 children suffered from injury anterior interosseous nerve and brachial artery lesion before fracture reposition. The coexistence of these complications was not associated with prolonged return of full function damaged nerve (return function after 22 days after the injury (3–34 days)).

**Figure 2:**
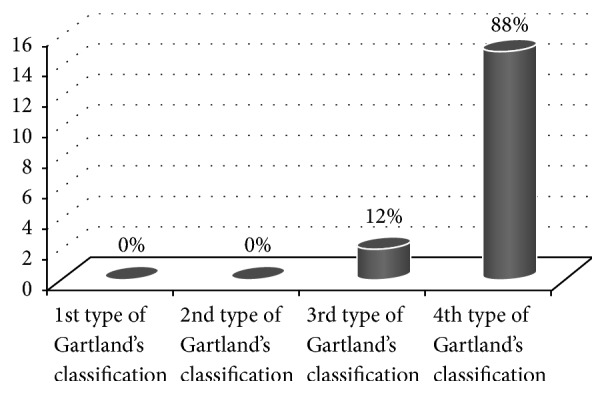
Type of fraction according to Gartland and the incidence of vascular damage.

**Figure 3:**
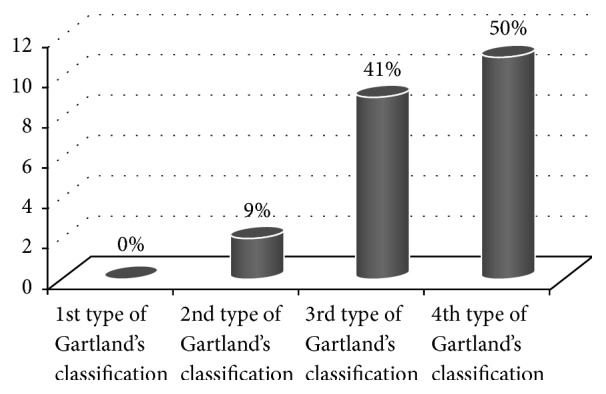
Type of fracture according to Gartland and the incidence of neurological complications.

Dependence of vascular injuries on the degree of fracture displacement is illustrated in Figure 2 and dependence of the neurological complications is presented in Figure 3.

According to it, even 88% of vascular and 50% of nerves injuries were accompanied by 4th type of Gartland's classification.

## 4. Discussion

During the diagnostic process of supracondylar fractures it is necessary to conduct physical examination to determine the location and the type of fracture, to assess the stability, and to detect early complications. To exclude early complications pulse should be examined on the radial and ulnar artery (eventually brachial artery). Assessment of warmth of the limb and capillary return and performing pulse oximetry are essential [11, 14, 15]. The neurologic examination must be also performed. It was suggested that detailed preoperative neuromuscular recording has to be done to avoid unclear postoperative situations [16].

The basic test is additional X-ray examination of the elbow in the AP and lateral projections, eventually additional oblique projections [9].

Nondisplaced fractures (type I fractures) should be managed in a long arm cast with the elbow in approximately 60 to 90 degrees of flexion for approximately three weeks. The most common method of treatment of displaced fractures of the corresponding types II, III, and IV according to Gartland/Leitch is closed reduction and percutaneous Kirchner wire stabilization [9, 17]. But sometimes (2,6%) the open fracture reduction is required [18].

Lateral-entry pin fixation is the management of supracondylar fractures in children [3, 4]. In the study of mathematical model, we can see that the cross-pin fixation (one wire from the lateral entry and the other one from medial side) provides better fixation strength [19–22].

In the clinical practice, it appeared that this method includes the possibility of iatrogenic ulnar nerve injury [23] and therefore is rarely used. In most cases, the lateral stability with 2 or 3 wires is sufficient [4].

In cases of incomplete reduction and in the case of open fractures and vascular damage, open reposition using minimally invasive techniques [1] or with full access [5, 10] should be considered. Supracondylar fractures of the humerus are quite frequently accompanied by various complications. Early complications, which occur immediately after the injury, include nerve and vascular injuries, muscular damage, and compartment syndrome. Cubitus varus, valgus deformity, hyperextension, restriction or lack of motion in the elbow, pin track infections, and compartment syndrome are late complications [4, 24, 25].

Frequency of acute nerves injuries accompanying supracondylar humeral fractures in children in different studies ranges from 10 to 20% [4, 6, 8, 14, 15, 22]. According to them, the most often complication is median nerve injury and anterior interosseous nerve injury [4, 8, 26]. We obtained a similar result in our work.

We have to notice the distinction between the median nerve damage and the pseudoanterior interosseous neuropathy [13].

Anterior interosseous nerve is a branch of the median nerve, which contains mostly motor fibers innervate muscles: a flexor pollicis longus, a flexor digitorum profundus of the index finger, and the pronator quadratus. Damage of this nerve revealed weakness/unnatural extension of the distal interphalangeal joint of the index finger and interphalangeal joint of the thumb. A characteristic feature is the lack of sensitivity [13, 26]. To assess this, you can use three tests: a hand clenching, making the “OK” sign [13, 26], and the picking up a coin from the ground by typing the thumb and index finger. In addition to motor nerve branches anterior interosseous nerve gives sensory branches to the wrist [13] but it is not clinically important.

Isolated nerve injuries resulting from supracondylar humeral fractures in the vast majority are treated conservatively [4, 27]. In most cases, symptoms disappear after 6 months without further surgery [4]. However, in cases where the symptoms of nerve injuries occurred after closed or open reduction, in the presence of an inadequate/incomplete reduction or strong, persistent neuropathic pain, when nerve function was completely suppressed and ischemia coexists, an open surgery and nerve exploration should be considered [27]. However, some authors present permanent nerve injury after treatment and the ulnar nerve injury after medial pin fixation was the most common [12, 16].

Arterial damage reported in the literature occurs with a frequency of 3.2%−14.3% [11, 14, 15, 28–30]. Brachial artery lesion may be secondary to various insults, such as entrapment, division, spasm of the vessel, the presence of an intimal tear, or thrombus formation [11]. Among the authors there are differences in the regulation of using the additional diagnostic tests such as ultrasound, angiography, CT, MRI angiography, and electromyography and their relevance in cases of supracondylar fractures [7, 8, 11, 29, 31]. Implementation of angio-CT and angio-MRI is not recommended in cases of supracondylar fracture, accompanied by absence of pulse in the radial artery [4, 11, 20]. Angiography is not indicated for a pulseless limb. This study does not affect the outcome of treatment and delays the time of fracture reduction. Closed reposition routinely used in this type of injury usually restores the pulse [4, 20]. It seems that angiography but especially ultrasound may be helpful in monitoring the conservative treatment to detect early signs of neuromuscular damage and circulatory disorders [1, 11, 31]. In many studies it turned out, however, that angiography and ultrasound do not bring tangible benefits [8, 29, 32].

The vascular spasms can persist for 24–48 hours making an early vascular intervention in otherwise symptomless patients not necessary [11, 30].

Controversies also exist in treatment of complications accompanying fractures, such as in cases of the so-called “pink, pulseless hand,” while some authors consider that conservative treatment and systematic control are fully sufficient because the vascular spasms can persist for 24–48 hours, making an early vascular intervention in otherwise symptomless patients not necessary [7, 11, 14, 28, 30, 33] and even that early intervention by a vascular surgeon to repair the brachial artery is associated with a high rate of reocclusion and residual brachial artery stenosis [7, 28, 34]. Others recommend aggressive surgical treatment and reconstruction of damaged vessels [14, 32, 35], because reliance on collateral flow in the forearm may leave the hand viable and, however, puts the child at risk for long-term sequelae such as contractures and limb length discrepancy [11, 14]. In some studies, radical treatments with interposition of the greater saphenous vein or the basilic vein for reconstruction of the brachial artery obtained very good and promising results [14, 36]. In our opinion, each case should be considered individually.

In cases where the limb is cold and there are clear signs of poor perfusion, most authors agree that urgent intervention by vascular surgeon is required [4, 7, 11, 14, 29]. Also if a pulse was present preoperatively but is absent following reduction and pin fixation, immediate rereduction is indicated. In such cases it may be assumed that artery was entrapped in the fracture site [4], because untreated vascular injury could lead to cold intolerance, neurologic deficits, and muscle stiffness and in the end to Volkmann ischemic contracture [7, 11].

We have found 2,27% cases which do not confirm the above mentioned importance of correlation [11, 22].

We recognize the limitation of our study concerning the surgical treatment of the supracondylar humeral fractures with the neurovascular complications. In our patient's group we did not present the cases treated without immediate fracture reposition and stabilization or vascular radiological examinations before reposition. We agree with the majority opinion that types II–IV performed by an experienced operator protect the patient from late complications and result in rapid regression of early complications.

## 5. Conclusions

  

1. In children with supracondylar fracture the most often injured nerve is median nerve
2. The incidence of vascular and nerve complications positively correlates with the progression of fracture according to Gartland classification
